# Impact of Age and Body Mass Index on the Outcomes of Laparoscopic Mesh Sacrocolpopexy

**DOI:** 10.1155/ogi/1706041

**Published:** 2025-01-16

**Authors:** Nour Khalil, Nadine El Kassis, Malak Moubarak, Christian Chaccour, Samer Maalouf, Elie Nemr, Houssein El Hajj, Maroun Moukarzel, David Atallah

**Affiliations:** ^1^Department of Urology, Hotel Dieu de France Hospital, Beirut, Lebanon; ^2^Department of Gynecology and Obstetrics, Hotel Dieu de France Hospital, Beirut, Lebanon

**Keywords:** genital prolapse, laparoscopy, obesity, sacrocolpopexy

## Abstract

**Background:** Pelvic organ prolapse (POP) is a benign condition that can adversely affect women's quality of life. Mesh sacrocolpopexy is an effective surgical treatment for POP, but is considered a complex and risky surgery for obese and elderly women. The objective of this study was to assess the impact of age and obesity on the outcomes of minimally invasive sacrocolpopexy.

**Methods:** We performed a retrospective cohort study reviewing all minimally invasive sacrocolpopexy cases performed between 2003 and 2021. Data on operative time, hospital stay, conversion rate, perioperative injuries, early and late postoperative complications were collected. Surgical success was evaluated by gynecological examination at each follow-up visit.

**Results:** One hundred seventy subjects were included, of whom 44% were older than 65 years and 58% had a body mass index (BMI) above 25 kg/m^2^. Seventy percent presented stage III uterovaginal prolapse. All patients achieved a good subjective outcome with no reported prolapse with a mean follow-up of 6 years. The rate of de novo stress urinary incontinence was 3.2%. Vaginal implant exposure was found in 4% of cases. A bivariate analysis studying the impact of older age (≥ 65 vs. < 65 years) and higher BMI (≥ 25 vs. < 25) on surgical and postoperative outcomes did not show any significant differences between the subgroups (*p* > 0.05).

**Conclusion:** In experienced hands, laparoscopic sacrocolpopexy can be used as a safe and effective procedure for operable patients with POP, even when patients are between 65 and 80 years or have a BMI of 25 kg/m^2^ and above.

**Summary**

• This study aims to assess the impact of weight and age on the outcomes of patients undergoing laparoscopic sacrocolpopexy.

## 1. Introduction

Pelvic organ prolapse (POP) is a very common complaint in multiparous women, estimated between 3% and 11% in women over 40 [1–3]. Increasing age and body mass index (BMI), as well as increasing parity, constitute the main risk factors for pelvic floor dysfunction [4]. Due to the aging population and the increase in obesity worldwide [5], dealing with genitourinary prolapse in elderly and obese patients is expected to be a major public health burden in the coming years [6].

During the last decade and with the emergence of minimally invasive surgery, abdominal sacrocolpopexy is gaining popularity as the technique of choice for apical POP, due to its very encouraging long-term results [7]. However, laparoscopic surgery is more at risk of complications, especially in the elderly and obese [8]. It was shown that operative times are increased in the obese population, as well as hospital stays and operative morbidity [9].

In elderly patients, the vaginal route has long been preferred over the abdominal one, given the widespread opinion on the safety and rapidity of this approach, which makes it better tolerated than abdominal surgery [8]. However, there are few data on the morbidity of laparoscopy in the elderly population.

In this study, we will analyze a cohort of patients undergoing minimally invasive sacrocolpopexy to see whether increased age and BMI would lead to more morbidity and a higher recurrence rate.

## 2. Materials and Methods

A single-center retrospective study was conducted at our university hospital, between 2003 and 2021. This study was approved by the Ethics Review Committee of our institution (CEHDF973—April 2017). Along with admission papers, all included patients had filled a form indicating that they agree for their data to be used for research purposes.

Patients who underwent mesh laparoscopic sacrocolpopexy between January 2003 and December 2021 were included in the cohort. A standardized surgical technique was performed by two different surgeons experienced in urogynecological surgery: an anterior-posterior double arm sacrocolpopexy using a polypropylene monofilament mesh. The mesh used were Pro-swing-Textile Hi-Tec, Fr and PRO-Swing PS4—Balmer Medical, Fr.

Before surgery, all patients underwent a thorough clinical examination to assess POP, which was reported according to Baden Walker classification. Patients who were diagnosed with stress urinary incontinence (SUI), on physical exam (full bladder cough test after prolapse reduction), were offered the option to have a simultaneous trans-obturator sling (TOT).

All patients received antibiotic prophylaxis ad low molecular weight heparin during their hospital stay and for two postoperative weeks.

### 2.1. Surgical Technique

After general anesthesia, abdominal access was obtained with an open technique using the umbilical stalk. The patient is placed in Trendelenburg position and four trocars are inserted under direct vision in both iliac fossas and in the para rectal space. First step consists in identifying and preparing the sacral promontory for the fixation of the mesh.

**Figure 1:**
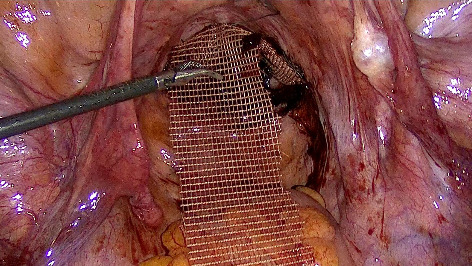
Posterior fixation of the mesh.

After opening of the posterior peritoneum medial to the right ureter, dissection of the recto vaginal space is undertaken until identification of both utero sacral ligaments and levator muscles. The posterior mesh is then sutured to the utero-sacral ligaments and levator muscles bilaterally with a final fixation at the level of the isthmus. Nonresorbable polyfilament suture 2-0 Ethibond is used for the sutures (Figure 1).

**Figure 2:**
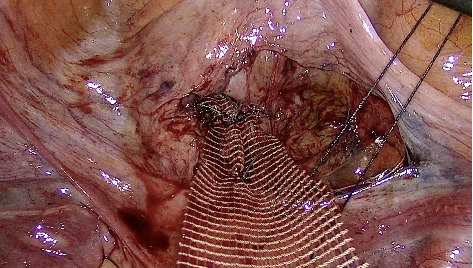
Anterior fixation of the mesh.

**Figure 3:**
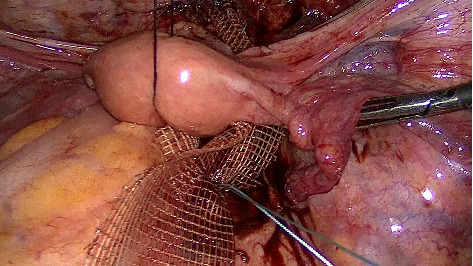
Double mesh fixation to the sacral promontory.

Then the anterior peritoneum is opened, and vesico-vaginal dissection is initiated and extended to the bladder neck. The anterior mesh is fixed to the anterior vaginal wall with separated sutures (Figure 2). The anterior and posterior meshes are attached to the anterior longitudinal sacral ligament, with an extracorporeal knot (Figure 3). The peritoneal windows are closed with a resorbable suture.

### 2.2. Acquisition of Data

The following characteristics were collected for each patient: age, BMI, parity, and grade of prolapse. Operative data collection included associated procedures performed (adhesiolysis, SUI surgery), operative time, conversion rate, estimated blood loss, perioperative incidents (urinary, digestive, or vascular injuries) and length of hospital stay (LOS). Postoperative follow-up was scheduled systematically for all patients at 5 weeks, 6 months and yearly thereafter. Surgical success was assessed by gynecological examination at each follow-up visit. Data on early (voiding difficulties, delayed mobility, wound complications, febrile morbidity, postoperative ileus, thromboembolic phenomena) and late postoperative complications (prosthesis-related, dyschezia, and constipation, dyspareunia) were collected, as well as the rates of POP relapse and the occurrence of de novo SUI. Data were collected from each follow-up visit to the clinic and from hospital visits (if presenting to the hospital emergency department).

### 2.3. Statistical Analysis

The distribution of age and BMI was considered parametric based on the histogram distribution and Q-Q plots, therefore, parametric tests were used for this study. To determine the appropriate threshold for age and BMI according to postoperative complications, a ROC analysis was performed. The area under the curve (AUC) was very low to establish the age and BMI cut-offs. Accordingly, the median was used as a cutoff, and patients were divided into age and BMI groups, using thresholds of 65 years and 25 kg/m^2^, respectively.

Operative and postoperative parameters were compared between the age and BMI groups, using the ki2 test or the student's test for qualitative or quantitative data, respectively. Pearson's correlation test for continuous variables was performed between operative time, hospital stay length, prolapse grade with BMI and age, respectively. The level of significance was set at 5%. Statistical analysis was performed using IBM SPSS Statistics (version 25).

A follow-up curve was established that describes how many patients were still present in postoperative follow-up.

## 3. Results

**Figure 4:**
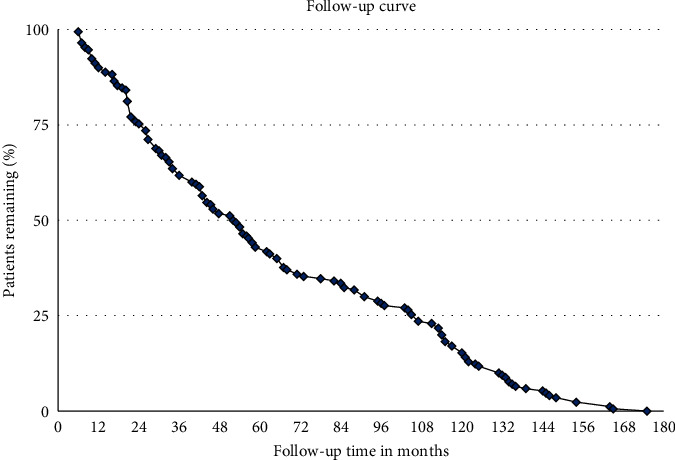
Distribution of patients according to last follow-up.

A total of 170 patients were included in the study. Demographic parameters are reported in Table 1. The duration to last follow-up is reported in Figure 4.

All patients were suitable for general anesthesia and their American Society of Anesthesiologists (ASA) score ranged between 1 and 3. Patients 65 years and older had no cognitive impairment or decreased functional status.

Most of the patients (70%) had Grade 3 POP compared to 6% and 22% for Grade 2 and Grade 4, respectively. Twenty-five percent underwent concomitant TOT placement for documented SUI.

Six patients benefited from placement of only the anterior mesh while all other patients underwent an anteroposterior fixation. All patients had a uterus, and none had a concomitant hysterectomy.

Blood loss was estimated for all patients less than 200 mL, and transfusion was never necessary. Adhesiolysis was necessary in 9% of the cases. Patients with adhesiolysis did not require a longer hospital stay (*p*=0.3). No conversion was performed for any of the patients. Operative time, LOS, and duration of follow-up are reported in Table 1.

Three per-operative complications (1.8%) were reported:

- Vesical breach sutured with 3-0 vicryl. The Foley catheter was left in place and removed after 7 days.
- Utero-ovarian bleeding and hematoma that led to hemostatic right adnexectomy.
- Rectal serosa lesion, closed with reinforced 2-0 vicryl sutures and verification of etancheity with rectal insufflation (by a colorectal surgeon).

During follow-up, patients complaining of bulging sensation, sexual, urinary, or bowel dysfunction were examined, and overall satisfaction was assessed. Anatomical recurrence was assessed by the Valsalva maneuver on pelvic gynecologic exam, and no prolapse recurrence was detected at follow-up.

The overall rate of complications was estimated at 11% (including early and late postoperative complications). The rate of de novo SUI that required TOT reoperation was 4%.

Prosthesis related complications consisted in vaginal exposure of the implant and was diagnosed in 4 patients (2%): 2 patients were classified as 2A T4 S1, and the other 2 as 2B T4 S1 (discharge) [10].

Differences according to age (< 65 and ≥ 65 years) and BMI (< 25 and ≥ 25 kg/m^2^), between operative time, hospital stay, and rate of complications are reported in Table 2. No statistical differences were found for all parameters between the groups.

Correlation tests performed between operative time, hospital stay, edge of prolapse with BMI and age, respectively, did not show any statistical significance (Table 3).

## 4. Discussion

After reviewing surgical alternatives for POP management, several reports have demonstrated that mesh sacrocolpopexy is the ideal technique in terms of objective and subjective success rates on long term follow-up [7], with better outcomes compared to native tissue vaginal repairs and vaginal sacrospinous fixation [11–14]. Anterior and posterior suspension is the recommended approach to avoid the retreading effect that occurs when the reinforcement of one compartment reveals the weakness of another [15].

According to the American College of Obstetricians and Gynecologists (ACOG) guidelines, mesh sacrocolpopexy is currently recommended for patients with shortened vaginal length, intra-abdominal pathology, or risk factors for recurrent POP (age less than 60 years, stage 3 or 4 prolapse, and BMI greater than 26) [16, 17]. In practice, however, and with the recent emergence and development of minimally invasive surgery, it is performed more frequently due to encouraging long-term results, even in severe prolapses.

The short-term complications that followed this procedure are minor ones. Using the Clavien–Dindo classification, the early postoperative complications were mostly Grades 1 and 2, without Grade 4 or 5 complications [18].

- Vesical breach: Vesical dissection can be very tricky in unexperienced hands, especially with severe cystoceles. Fortunately, small breaches can be efficiently repaired laparoscopically, and the mesh can be inserted, nonetheless.
- Rectal serosa breach: Although rectal injuries counter-indicate mesh insertion due to contamination of the operative field, we only had a slight serosal breach; therefore, we proceeded with mesh insertion.

Our data demonstrated an excellent success rate at a mean follow-up interval of 6 years, with a lower rate of prosthesis-related complications compared to the rates mentioned in the literature (2% vs. 4.2%) [11].

It is known that combining prolapse and incontinence surgery may include increased operative complexity, higher risk of complications, extended recovery time, and discomfort. However, in our cohort, we did not find any increase in morbidity for patients undergoing combined TOT and prolapse surgery.

Obese patients are known to have a high risk of relapse and progression of uterovaginal prolapse compared to women with healthy BMI. Performing mesh sacrocolpopexy for obese patients has been thought to increase blood loss during surgery with a prolongation in operating times [19], and was associated with more postoperative complications in the early postoperative period [20]. Our results did not show significant differences in terms of operating time between the normal weight and the overweight group. Furthermore, obese patients in our series were not at increased risk of operative injuries and postoperative complications. Conversion to laparotomy was not an issue in our series (0 cases), and this was attributed to the performance of an open laparoscopic technique (using the umbilical stalk), with uterine and sigmoid suspension that facilitates exposure throughout the procedure. The patients were operated by highly experienced surgeons, which explains why operating times, conversion rates, injuries, and therefore early complications were not affected by the BMI of the patient.

When considering the age of patients, since mesh sacrocolpopexy is the recommended procedure for younger women, operating elderly patients using the same technique seems feasible if the complication rates are comparable between the two groups. Since an increase in life expectancy is expected in the future, using the most efficient technique would allow elderly patients to achieve better and more durable outcomes, therefore decreasing recurrences and avoiding reinterventions for vaginal vault prolapse at very old ages where operative risks are multiplied and operability is questionable [21]. Using an effective technique for sacrocolpopexy, favorable and durable outcomes can be achieved. Consequently, this approach helps prevent recurrence and the need for reoperation in elderly patients.

In our series, we have demonstrated the safety of mesh sacrocolpopexy in operable elderly patients, without an increase in the risk of early postoperative complications and LOS. It has previously been thought that age is a predictor of complications in the postoperative period, especially when surgery is expected to be complex and lengthy, such as minimally-invasive sacrocolpopexy [22, 23]. However, when surgery is performed in centers with a high urogynecological workload and with experienced hands, the safety of the procedure is maintained [24].

In practice, age and BMI should not be regarded as an obstacle to sacrocolpopexy anymore. Surgeons with common laparoscopic practice should be at ease performing this procedure to correct even severe prolapses. Moreover, this study showed no specific problems related to the use of prosthesis for the prolapse correction, even on the long term.

The main limitation of our study is its retrospective nature. However, the fact that the data were collected prospectively limits the recall bias of the study. Another limitation would be the fact that some patients would have followed up elsewhere on the long term, and therefore maybe late complications are missing.

## 5. Conclusion

This study showed that mesh sacrocolpopexy is associated with high success rates, good functional results, and low rates of surgical complications, when performed by experienced surgeons, even in patients with BMI over 25 and age over 65. These results should be reassuring for clinicians treating overweight and elderly patients presenting for POP. Also, based on these results, there is no clear morbidity associated with the use of prosthesis for reduction of urogenital prolapse, even on the long term.

## Figures and Tables

**Table 1:** Demographic and operative parameters.

	Mean ± SD	Median [IQR]
Demographics		
Age (years)	61 ± 11	63 [55–69]
Parity	3.8 ± 1.8	4 [3–5]
Body mass index (kg/m^2^)	25 ± 4	25 [18–39]
Operative parameters		
Operative time (min)	163 ± 39	180 [120–180]
Length of hospital stay (days)	3.3 ± 1.8	3 [2–4]
Duration of follow-up (months)	64 ± 47	52.5 [25.5–107]

**Table 2:** Operative outcomes between age and body mass index groups.

Age (years)	< 65	≥ 65	*p* value
Hospital stay (days)	3.3	3.2	0.20
Operative time (min)	161	166	0.50
Per-operative incident	1.4%	2.2%	0.99
Complication	6.7%	14.7%	0.10
Vaginal exposure	4.2%	0.0%	0.13

**Body mass index (**kg/**m**^2^**)**	**< 25**	**≥ 25**	**p** **-value**

Hospital stay (days)	3.37	3.29	0.71
Operative time (min)	162	164	0.96
Per-operative incident	0.0%	2.5%	0.51
Complication	11.9%	9.8%	0.69
Vaginal exposure	1.7%	3.7%	0.64

**Table 3:** Correlation between length of hospital stay, operative time, grade of prolapse with body mass index and age.

		Hospital stay	Operative time	Grade prolapse
Age	Pearson	−0.09	0.09	−0.1
	*p* value	0.24	0.26	0.30

Body mass index	Pearson	−0.02	−0.02	0.31
	*p* value	0.76	0.75	0.31

## Data Availability

Used data can be shared if requested.

## Nomenclature

BMI: Body mass index
POP: Pelvic organ prolapse
SUI: Stress urinary incontinence
TOT: Tension-free vaginal tape
UUI: Urge urinary incontinence
